# Cryoballoon-Based Left Atrial Appendage Isolation and Closure in Patients with Atrial Fibrillation—The LALALAND Pilot Study

**DOI:** 10.3390/jcm15082980

**Published:** 2026-04-14

**Authors:** Christian-H. Heeger, Samuel Reincke, Sorin Stefan Popescu, Sascha Hatahet, Behnam Subin, Anna Traub, Karl-Heinz Kuck, Charlotte Eitel, Roland R. Tilz

**Affiliations:** 1 University Heart Center Lübeck, Department of Rhythmology, University Hospital Schleswig-Holstein, 23538 Lübeck, Germany; christian.h.heeger@gmail.com (C.-H.H.);; 2 German Center for Cardiovascular Research (DZHK), Partner Site Hamburg/Kiel/Lübeck, 23538 Lübeck, Germany; 3 Department of Rhythmology, Asklepios Klinik Hamburg Altona, 22763 Hamburg, Germany; 4 FESC, FEHRA, FHRS, Klinik für Rhythmologie, Universitäres Herzzentrum Lübeck, Universitätsklinikum Schleswig-Holstein (UKSH), Ratzeburger Allee 160, 23538 Lübeck, Germany

**Keywords:** atrial fibrillation, pulmonary vein isolation, cryoballoon, acute efficacy, follow-up

## Abstract

**Background**: Atrial fibrillation (AF) remains the most common cardiac arrhythmia, with pulmonary vein isolation (PVI) established as the cornerstone of interventional treatment. However, in patients with persistent AF (PersAF), the success rates of PVI alone tend to be limited. A promising additional target is the left atrial appendage (LAA). In recent years, cryoballoon (CB) technology has become a tool for achieving durable PVI. Its application for LAAI has been investigated as a potentially advantageous alternative to radiofrequency ablation, and a positive effect on long-term outcome has been reported. However, the available data is limited. This study sought to investigate the clinical impact of CB-based LAAI in addition to PVI. **Methods:** This is a prospective, interventional, single-centre study. Consecutive patients with symptomatic PersAF were prospectively enrolled. In total 23 patients with PersAF underwent PVI plus LAAI using the CB system. Percutaneous LAA closure was performed within 2–3 months in all patients by implanting an endocardial LAA-closure device. Prior to LAA closure, LAAI durability was systematically assessed by invasive remapping studies. **Results:** A total of 100% of PVs were successfully isolated using the CB only (n = 91/91). Concerning LAAIs, a total of 21/23 (91%) remained isolated at the end of the procedure. After the ablation procedure including LAAI, all patients were scheduled for TEE assessment and LAA closure. TEE was performed after a mean of 54 ± 19 days. In 6/23 (26%) patients, LAA thrombus formation was detected after LAAI. A total of 23/23 patients (100%) received LAAC after a mean of 72 ± 45 days. Durability of LAAI was assessed utilizing a spiral mapping catheter in 23/23 patients (100%). In a total of 17/23 (74%) patients, durable LAA isolation was detected. Durable PVI of all PVs was detected in 16/23 (70%) patients. During a mean follow-up of 13 ± 3.4 months, stable sinus rhythm was maintained in 15 (65%) patients. The LAA showed reconnection in 3/23 (13%) patients, with arrhythmia recurrence. During follow-up, one stroke (318 days after LAAC) and one device thrombus (56 days after LAAC) occurred. **Conclusions:** While CB-based LAAI may offer benefits in managing persistent AF, it presents a significant risk of thrombus formation in the LAA, even with appropriate OAC. Early closure of the LAA following LAAI appears promising in mitigating these risks, but further evidence is needed to establish clear best practices.

## 1. Introduction

Atrial fibrillation (AF) remains the most common sustained cardiac arrhythmia, with pulmonary vein isolation (PVI) established as the cornerstone of interventional treatment, especially for paroxysmal AF [1,2,3]. However, in patients with persistent AF (PersAF), the success rates of PVI alone tend to be limited, prompting the exploration of additional ablation strategies. One promising target that has garnered increasing attention is the left atrial appendage (LAA) [4,5,6,7,8]. Recent studies suggest that electrical isolation of the LAA may enhance procedural success by addressing potential arrhythmogenic substrates outside the pulmonary veins [8]. Traditionally, LAA isolation (LAAI) has been performed using radiofrequency (RF) energy, but this method carries risks such as electromechanical dissociation, which may increase the likelihood of thrombus formation and subsequent thromboembolism—even under oral anticoagulation (OAC) therapy [4,5]. In recent years, cryoballoon (CB) technology has become a prominent tool for achieving durable PVI due to its safety and efficacy profile [9]. Its application for LAAI has been investigated as a potentially advantageous alternative to radiofrequency ablation, and a positive effect on long-term outcome in PersAF patients has been reported [10]. The CB’s advantages include simplified application and consistent lesion formation, which could translate into more-reliable LAAI. However, the safety, long-term efficacy, and impact on thromboembolic complication rates of CB-based LAAI are still being evaluated. As such, this approach represents a promising, minimally invasive strategy that could improve outcomes in patients with persistent AF, while potentially reducing thromboembolic risks associated with traditional methods. However, the available data is limited, varies across a wide range and lacks long-term assessments. This study sought to investigate the clinical impact of CB-based LAAI in addition to PVI, the incidence of postprocedural LAA thrombus formation and thromboembolism as well as the impact of interventional LAA closure.

## 2. Methods

### 2.1. Study Design and Patient Population

This is a prospective, interventional, single-centre study. Consecutive patients with symptomatic PersAF were prospectively enrolled. In total 23 patients with PersAF underwent PVI plus additional LAAI using the Arctic front advance (CB2) or Arctic front advance pro (CB4) CB system. Exclusion criteria were prior left atrial (LA) ablation procedures, a left atrial (LA) diameter >60 mm, severe valvular heart disease or contraindications to postinterventional oral anticoagulation. All patients provided a written informed consent before inclusion. All patient-related data were anonymized. The LALALAND Pilot study was approved by the local ethics committee (Lübeck ablation registry ethical review board number: WF-028/15) and was performed in accordance with the ethical standards laid down in the 1964 Declaration of Helsinki and its later amendments. Primary study endpoints were postprocedural LAA thrombus formation, thromboembolic events, and clinical success in terms of 12-months freedom from AF or atrial tachycardia recurrence. Secondary endpoints were procedure-related complications and assessment of the prevention of postprocedural thromboembolic events.

#### 2.1.1. Preprocedural Management

Preprocedural transoesophageal echocardiography (TEE) was performed to rule out intracardiac thrombi before the procedure. No additional preprocedural imaging was carried out. In patients under treatment with vitamin K antagonists (VKAs), the procedure was conducted under therapeutic international normalized ratio (INR) values between 2 and 3, while in those under non-VKA oral anticoagulants (NOACs), the morning dose of NOAC was omitted on the day of the procedure [11].

#### 2.1.2. Intraprocedural Management

All procedures were performed by two physicians, who were highly experienced in CB procedures. A comprehensive description of the intraprocedural management has been reported in previous publications from out department [6,11]. Briefly, the procedures were performed under deep sedation using midazolam, fentanyl and continuous infusion of propofol. Two ultrasound-guided right femoral vein punctures were carried out, and two 8-French (F) short sheaths were inserted. One 7 F diagnostic catheter was positioned within the coronary sinus (CS) via a sheath in the right femoral vein. Single transseptal puncture (TSP) was performed under fluoroscopic guidance using a modified Brockenbrough technique and an 8.5 F transseptal sheath (SL1; St. Jude Medical, Inc., St. Paul, MN, USA). After the TSP, LA access was confirmed by contrast medium injection via the transseptal needle. A selective angiography of all PVs was carried out utilizing a 7 F multipurpose catheter or the transseptal sheath, to identify the PV ostia. Afterwards, the 15 F Flexcath Advance sheath (Medtronic, Inc., Minneapolis, MN, USA) was inserted over a guidewire in the transseptal position. Both sheaths were continuously flushed with heparinized saline (20 mL/h). After the TSP, an activated clotting time (ACT) of >300 s was targeted by means of heparin boluses.

### 2.2. Cryoballoon PVI

The intraluminal oesophageal temperature was monitored during each refrigerant injection using a spiral multi-site oesophageal probe (CIRCA S-CATH; Circa Scientific, Englewood, CO, USA). An intraluminal oesophageal temperature cut-off value of 15 °C was used to avoid oesophageal thermal injuries. In case of a temperature drop <15 °C, the application was stopped immediately using a double-stop technique [12,13,14].

During the isolation of the septal pulmonary veins, continuous phrenic nerve (PN) pacing using a maximum output and pulse width at a cycle length of 1000–1200 ms through a 7 F diagnostic catheter positioned in the superior vena cava was performed. Intermittent fluoroscopic evaluation, tactile feedback of the contractions of the diaphragm and continuous motor action potential (CMAP) monitoring were used to assess the PN capture [15,16]. Weakening or loss of diaphragm movement or a reduction in the CMAP amplitude of at least 30% led to an immediate termination of energy delivery using the double-stop technique [17]. In case of PN palsy, no additional energy delivery was applied at the level of the right PV.

The ablation sequence started with the left superior PV (LSPV), followed by left inferior PV (LIPV), right inferior PV (RIPV) and right superior PV (RSPV). Afterwards the LAA was targeted. Before initiating the freezing cycle, the PV occlusion was verified by contrast medium injection under fluoroscopy. Additionally, a second injection was performed 5–10 s after starting the freezing cycle to assess the stability of the occlusion. If the stable occlusion was confirmed, the freezing cycle was continued, otherwise the balloon was repositioned, and a third contrast medium injection was used to reassess the occlusion, or the freezing cycle was stopped, and another ablation attempt was made. After 70 s of freezing, a gentle pull-down manoeuvre was performed during isolation of the inferior PVs in all cases. For PVs, a no-bonus freeze protocol with 180 s of freezing time was utilized. The procedural PVI’s success was defined by the disappearance of all PV recordings on the spiral mapping catheter positioned inside the PVs after energy delivery (entrance block). Further pacing manoeuvres and adenosine testing were not performed.

After verified isolation of all PVs the LAA was targeted and electrically isolated as described earlier by our group [6,7]. The application time was prolonged to 300 s. After proven LAAI, a bonus freeze application of 300 s was applied, and LAAI was confirmed by entrance block. Left phrenic nerve stimulation was performed via the Achieve catheter placed in the LAA during the CB applications to detect and prevent injury of the left phrenic nerve. An electrical cardioversion was carried out after the final freezing cycle to achieve sinus rhythm (SR), if necessary.

### 2.3. Postprocedural Management and Follow-Up

For patients on preprocedural oral anticoagulation (OAC) therapy with phenprocoumon, the medication was not changed and was prolonged according to the previous prescription. For patients on preprocedural novel OAC medications, the first dose was prescribed 6 h after sheath removal at half the recommended dose. The full-dose novel OAC was started the following day. Life-long OAC therapy was strongly recommended to all patients. Surface ECGs, transthoracic echocardiography, and 24 h Holter ECG recordings were performed in our outpatient clinic or by the referring physician on day one postprocedure and repeated at 1, 3, 6 and 12 months.

### 2.4. Left Atrial Appendage Closure

Percutaneous LAA closure was performed within 2–3 months in all patients by implanting an endocardial LAA-closure device according to the instructions for use. A TEE examination was performed prior to LAA closure to evaluate LAA flow velocity and assess spontaneous LAA echo contrast (“smoke”) or thrombus. In case of LAA thrombus, OAC therapy was intensified to a target INR of 2.5–3.5 if phenprocoumon was used. When novel OACs were used, a switch to phenprocoumon with a target INR between 2.5 and 3.5 was recommended, and repeat TEEs were initiated until the thrombus disappearance. Prior to LAA closure, LAAI durability was systematically assessed by invasive remapping studies utilizing a circular mapping catheter. The utilized LAA-closure devices were Watchman Flx (Boston Scientific, Marlborough, MA, USA), LAmbre (Lifetech, Shenzhen, China) and Amulet (Abbott, Abbott Park, IL, USA).

### 2.5. Statistical Analysis

All analyses were performed using STATA software version 14.0 (STATA Corp, Lake Drive Way, TX, USA). Distributions of continuous variables were tested for normality using the Shapiro–Wilk test. Continuous variables are expressed as mean ± standard deviation (SD). Categorical variables are reported as counts (percentage). Comparisons of categorical variables were performed using chi-squared or Fisher’s exact test, as appropriate. All *p*-values reported are two-sided, and a *p*-value < 0.05 was considered statistically significant.

## 3. Results

### 3.1. Patient Characteristics

In total, 23 consecutive patients with predominantly clinical symptomatic PersAF were prospectively enrolled into the study. The median age was 70 (60.5, 70.0) years. Detailed patient baseline characteristics are shown in Table 1. A total of 11/23 patients previously reported LAA thrombus formation, which was not present at the time of the ablation procedure.

### 3.2. Catheter Ablation During the Index Procedure

The CB2 was utilized in 10/23 cases, while in 13/23 cases the CB4 was utilized. A total of 100% of PVs were successfully isolated using the CB only (n = 91/91). Periprocedural characteristics are depicted in Table 2.

Concerning LAAIs, a total of 21/23 (91%) remained isolated at the end of the procedure. Furthermore, eight (35%) LAAI patients were transient after initial isolation, while in two (8%) patients an isolation of the LAA was not achieved despite multiple attempts. A mean of 2.1 ± 1.4 CB applications was necessary to achieve LAAI, and a mean of 3.0 ± 1.4 applications was performed in total including a bonus freeze application. The mean minimal temperature was −52.8 ± 6.1 °C. The mean time to first LAAI was 209 ± 166 s, while the mean time to LAAI after initial LAAI and recovery was 130 ± 61 s in a total of eight patients with recovery of LAA conduction and a further attempt of LAAI. The total freezing time was 784 ± 434 s. A total of 1/23 patients experienced periprocedural complications. One patient experienced a transient left PN palsy (2%). No stroke or TIA was reported by any patients between LAAI and LAAC. Postprocedural OAC is displayed in Table 3.

### 3.3. Assessment of Left Atrial Appendage and Closure

After the ablation procedure including LAAI, all patients were scheduled for TEE assessment and LAA closure. TEE was performed after a mean of 54 ± 19 days. In 6/23 (26%) patients, LAA thrombus formation was detected after LAAI. TEE assessments and LAAC procedural findings are depicted in Table 4. Thrombus formation within the LAA was observed despite OAC with Apixaban (n = 4/6, 66%) or Warfarin (n = 3/6 33%). After changing the OAC regimen, LAA thrombi were resolved in 6/6 cases (100%). A total of 23/23 patients (100%) received LAAC after a mean of 72 ± 45 days. Durability of LAAI was assessed utilizing a spiral mapping catheter in 23/23 patients (100%). In a total of 17/23 (74%) patients, durable LAA isolation was detected. Durable PVI of all PVs was detected in 16/23 (70%) patients.

In terms of periprocedural complications during the LAAC procedure, one pericardial effusion without necessity of epicardial puncture was observed. One device dislodgement occurred a few hours after implantation. The device was retrieved, and a new implantation attempt was successfully performed. No stroke or TIA occurred periprocedurally. Postprocedural assessment of LAAC was performed utilizing TEE and is depicted in Table 5.

### 3.4. Clinical Outcome and Arrhythmia Recurrence After LAAI

**Figure 1:**
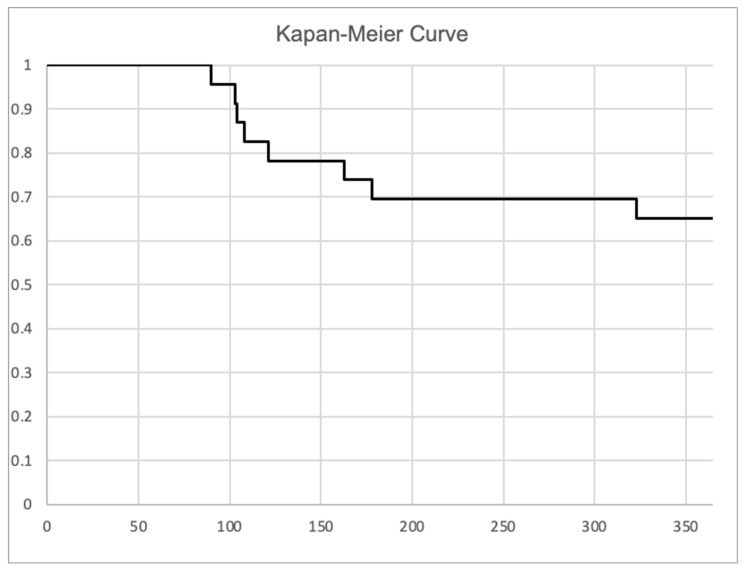
**Long-term recurrence-free survival.** The Kaplan–Meier curve demonstrates freedom from recurrence of atrial arrhythmias.

During a mean follow-up of 13 ± 3.4 months, stable sinus rhythm was maintained in 15 (65%) patients (Figure 1). The LAA showed reconnection in 3/23 (13%) patients, with arrhythmia recurrence. In patients with LAA thrombus after LAAI, 6/6 (100%) showed durable LAAI. In patients without durable LAAI, the rate of thrombus was 0/6 (0%) (*p* < 0.001). During follow-up, one stroke (318 days after LAAC) and one device thrombus (56 days after LAAC) occurred. Both patients had durable LAAI and continued OAC, despite LAAC.

## 4. Discussion

The main findings of this prospective study are as follows: (1) A CB-based LAAI in addition to PVI seems to be safe and feasible. (2) The incidence of thromboembolic events and LAA thrombus and CB-based LAAI is substantially high despite sufficient OAC. (3) The rate of durable LAAI at follow-up was 74%. (4) CB-based LAAI in combination with percutaneous LAAC 2–3 months later did not result in an increased risk for thromboembolic events.

### 4.1. Left Atrial Appendage Thrombus Formation

The incidence of TEE-detected LAA thrombi has been described to be 0.24–5% and seems to be associated with LAA morphology. In the present study, we found LAA thrombi at a remarkably high rate of 26% after CB-based LAAI despite sufficient OAC. A lower LAA flow velocity and smoke before the LAAI and differences afterwards support the mechanism of LAA thrombus formation by local stasis within the LAA following electromechanical dissociation after LAAI. The latest findings on the incidence of LAA thrombus formation after LAAI are controversial. While in the BELIEF trial (RF-based circular LAAI) an incidence of 1.8% of LAA thrombus after LAAI has been observed, two studies by the Hamburg group detected high rates of thrombus formation after RF-based wide-area LAAI [4,5]. Interestingly previous findings on CB-based LAAI found no LAA thrombi, while in our recent study the rate of LAA thrombus formation was 26%.

Although different ablation strategies might be a possible explanation for this discrepancy, the underlying mechanism of electrical LAAI with consecutive loss of mechanical contraction should occur with all different techniques. Interestingly, in patients with durable LAAI, the incidence of thrombus within the LAA was higher than in patients without durable LAAI, which further supports the role of a permanently isolated LAA in thrombus formation. Potentially a higher dosage of OAC might prevent LAAI; however it increases the bleeding risk and is therefore not applicable.

### 4.2. Management of Thrombus Formation and LAA Closure After LAAI

Loss of LAA mechanical function after LAAI was suggested to potentially increase thromboembolic risk; therefore it seems reasonable to recommend life-long OAC after LAAI regardless of the CHA_2_DS_2_-VASc score. Due to the fact that >90% of patients with LAA thrombus were on sufficient OAC, this strategy seems not to be sufficient to prevent LAA thrombus and thromboembolic events. In our study no postprocedural stroke or TIA occurred between CB-based LAAI and LAAC.

LAA closure was strongly recommended to all patients after LAAI. The present data indicate that closure of the LAA following CB-based LAAI may be a viable option to reduce the risk of thromboembolism without the necessity to establish intensified OAC and its potential consequences. Therefore, we recommended that LAA closure or ligation should be performed after LAAI as early as possible due to potential risk in clinical practice.

### 4.3. Clinical Benefit in Long-Term Follow-Up

Creation of linear lesions and complex fractionated atrial electrogram ablation has been widely used for substrate modification in PVI non-responders. However, the benefit of these strategies is still controversial. The BELIEF trial showed that an empirical LAAI in addition to PVI is able to improve clinical success [9]. With evidence of improved clinical success after wide-area LAAI, our findings further support this observation. However, the optimal strategy for LAAI is still unclear, and the findings of both studies, especially concerning LAA thrombus and thromboembolic risk, need to be evaluated in further trials. Since RF-based wide-area LAAI as well as circumferential LAAI is often time consuming and increases the risk of complications, a CB-based LAAI could be performed with a relatively high acute success rate with a mean of 3.0 ± 1.4 CB applications (inclusive of a bonus freeze application) and an acceptable procedure time of 81.9 ± 30.9 and a low rate of periprocedural complications (4%, only one transient phrenic nerve injury). The 12-month rate of freedom of AF was 65% of patients, which is in line with recent findings of PVI without LAAI. The recently published ASTRO-AF study failed to show the benefit of CB-based LAAI in patients with durable PVI; however the patient populations are different in the ASTRO-AF study (repeat procedures with durable PVI) and the LALALAND pilot study (de novo PVI plus LAAI). Therefore, the clinical benefit of this strategy is still controversial, and randomized controlled trials are necessary to further analyse and investigate the strategy (LALALAND-AF study, ClinicalTrials.gov ID NCT04240366).

### 4.4. Limitations

The number of patients is low, and this single-centre experience was not randomized. Follow-up was limited to regular Holter ECGs and surface ECGs. This limitation might potentially overestimate the rates of stable sinus rhythm due to a reduced detection of recurring asymptomatic arrhythmic episodes. Due to the fact the only a very limited number of patients received LAAI in addition to PVI, there is a high potential for selection bias in this pilot design. The expected outcome for PersAF patients treated with PVI only is not different to the latest findings.

## 5. Conclusions

While CB-based LAAI may offer benefits in managing persistent AF, it presents a significant risk of thrombus formation in the LAA, even with appropriate OAC. Our small study presents data that an early closure of the LAA following LAAI might mitigate these risks, but further evidence is needed to establish clear best practices.

## Figures and Tables

**Table 1:** Baseline characteristics.

Number of Patients	23
Age (years)	70 (60.5, 70.0)
Female sex	3 (13)
BMI > 28	13 (57)
Left atrial diameter (mL/m^2^)	30 (29, 32)
Persistent AF	21 (91)
Duration of AF (months)	28 (6, 54)
Arterial hypertension	16 (70)
Coronary artery disease	5 (26)
Congestive heart failure	4 (17)
Diabetes mellitus type II	2 (9)
Prior TIA/stroke	8 (35)
Prior LAA thrombus	11 (48)
CHA_2_DS_2_-VASc score	3 (2, 4)
HASBLED score	2 (2, 3)

Continuous data are summarized as median [25th and 75th percentiles]. Categorical data are presented as N (%). AF = atrial fibrillation, TIA = transient ischaemic attack.

**Table 2:** Periprocedural data—catheter ablation.

Number of PVs *	91
Utilizing of CB2	10/23 (43)
Utilizing of CB4	13/23 (57)
Number of isolated PVs *	91/91 (100)
Number of isolated LAAs (at end procedure)	21/23 (91)
Procedure duration (min)	81.9 ± 30.9
Fluoroscopy time (min)	19.8 ± 9.0
Contrast medium (mL)	87 ± 30
Heparin (IU)	14,235 ± 5596
Major complications	0 (0)
Vascular access complications	0 (0)
Pericardial tamponade	0 (0)
Stroke/TIA	0 (0)
Minor complications	1 (4)
Transient phrenic nerve injury (left)	1 (4)
Pericardial effusion	0 (0)
Minor vascular complications	0 (0)

Continuous data are summarized as means ± standard deviations. Categorical data are presented as N (%). * PV = pulmonary vein

**Table 3:** Procedural details—individual pulmonary veins and LAA.

Variable	
**RSPV:**	
Total CB cycles until PVI	1.45 ± 1.6
Minimal temp., (°C)	−46.0 ± 10.2
Minimal oesophageal temp., (°C)	35.4 ± 0.8
Duration of total freezing time, s	220 ± 107
**RIPV:**	
Total CB cycles until PVI	1.05 ± 0.5
Minimal temp., (°C)	−45.9 ± 6.4
Minimal oesophageal temp., (°C)	32.8 ± 3.5
Duration of total freezing time, s	204 ± 87
**LSPV:**	
Total CB cycles until PVI	1.1 ± 0.3
Minimal temp., (°C)	−46.3 ± 5.8
Minimal oesophageal temp., (°C)	32.7 ± 4.9
Duration of total freezing time, s	206 ± 69
**LIPV:**	
Total CB cycles until PVI	1.15 ± 0.5
Minimal temp., (°C)	−45.0 ± 6.3
Minimal oesophageal temp., (°C)	30.5 ± 6.7
Duration of total freezing time, s	216 ± 94
**LAA:**	
Total CB cycles until LAAI	2.1 ± 1.4
Total CB cycles	3.0 ± 1.4
Total LAAI recovery after initial isolation	8 (35)
Total LAAI	21 (91)
Minimal temp., (°C)	−52.8 ± 6.1
Minimal oesophageal temp., (°C)	34.1 ± 3.5
Time to LAAI, s	209 ± 166
Time to LAAI after recovery, s	130 ± 61
Live signal recordings during ablation	19 (83)
Duration of total freezing time, s	784 ± 434

**Table 4:** Left atrial appendage assessment and closure.

LAA Thrombus During Follow-Up	6/23 (26)
LAA thrombus under OAC	6/6 (100)
LAA flow before LAAI, m/s	0.47 ± 0.21
LAA flow after LAAI, m/s	0.27 ± 0.25
Patients with thrombus, OAC: Apixaban	4/6 (66)
Patients with thrombus, OAC: Warfarin	2/6 (33)
LAA thrombus resolved	6/6 (100)
LAA closure performed	23/23 (100)
Watchman Flx device	15/23 (65)
Amulet device	7/23 (30)
LAmbre device	1/23 (4)
Time from ablation to LAAC, days	72 ± 45
Assessment of durable LAAI	23/23 (100)
Durable LAAI	17/23 (74)
Durable PVI	16/23 (70)
Procedure duration (min)	53.6 ± 27.8
Fluoroscopy time (min)	8.3 ± 4.4
Contrast medium (mL)	71 ± 19
Heparin (IU)	9923 ± 2753
Major complications	1 (4)
*Vascular access complications*	0 (0)
*Pericardial tamponade*	0 (0)
*Stroke/TIA*	0 (0)
*Device dislodgement*	1 (4)
Minor complications	1 (4)
*Pericardial effusion*	1 (4)
*Minor vascular complications*	0 (0)

**Table 5:** Long-term assessment of left atrial appendage closure.

Long-Term Assessment of LAAC	
Time to LAAC control, days	80 ± 60
Thrombus formation on LAAC	1/23 (4)
Gaps (>5 mm)	0/23 (0)
Postprocedural stroke/TIA	1/23 (4)

## Data Availability

Non-digital data supporting this study are curated at the study centre of the Department of Rhythmology, University Hospital Schleswig-Holstein, Germany.
